# Polarity directed optimization of phytochemical and in vitro biological potential of an indigenous folklore: *Quercus dilatata* Lindl. ex Royle

**DOI:** 10.1186/s12906-017-1894-x

**Published:** 2017-08-03

**Authors:** Madiha Ahmed, Humaira Fatima, Muhammad Qasim, Bilquees Gul, Ihsan-ul-Haq

**Affiliations:** 1 Department of Pharmacy, Faculty of Biological Sciences, Quaid-i-Azam University, Islamabad, 45320 Pakistan; 2 Institute of Sustainable Halophyte Utilization, University of Karachi, Karachi, 75270 Pakistan

**Keywords:** Antileishmanial, Antioxidant, Cytotoxicity, Hep G2, Protein kinase inhibition, THP-1 leukemia cell line

## Abstract

**Background:**

Plants have served either as a natural templates for the development of new chemicals or a phytomedicine since antiquity. Therefore, the present study was aimed to appraise the polarity directed antioxidant, cytotoxic, protein kinase inhibitory, antileishmanial and glucose modulatory attributes of a Himalayan medicinal plant- *Quercus dilatata*.

**Methods:**

Total phenolic and flavonoid contents were determined colorimetrically and various polyphenols were identified by RP-HPLC analysis. Brine shrimp lethality, SRB and MTT assays were employed to test cytotoxicity against *Artemia salina* and human cancer cell lines respectively. Antileishmanial activity was determined using standard MTT protocol. Glucose modulation was assessed by α-amylase inhibition assay while disc diffusion assay was used to establish protein kinase inhibitory and antifungal spectrum.

**Results:**

Among 14 extracts of aerial parts, distilled water-acetone extract demonstrated maximum extract recovery (10.52% *w*/w), phenolic content (21.37 ± 0.21 μg GAE/mg dry weight (DW)), total antioxidant capacity (4.81 ± 0.98 μg AAE/mg DW) and reducing power potential (20.03 ± 2.4 μg/mg DW). On the other hand, Distilled water extract proficiently extracted flavonoid content (4.78 ± 0.51 μg QE/mg DW). RP-HPLC analysis revealed the presence of significant amounts of phenolic metabolites (0.049 to 15.336 μg/mg extract) including, pyrocatechol, gallic acid, catechin, chlorogenic acid, p-coumaric acid, ferulic acid and quercetin. Highest free radical scavenging capacity was found in Methanol-Ethyl acetate extract (IC_50_ 8.1 ± 0.5 μg/ml). In the brine shrimp toxicity assay, most of the tested extracts (57%) showed high cytotoxicity. Among these, Chloroform-Methanol extract had highest cytotoxicity against THP-1 cell line (IC_50_ 3.88 ± 0.53 μg/ml). About 50% of the extracts were found to be moderately antiproliferative against Hep G2 cell line. Methanol extract exhibited considerable protein kinase inhibitory activity against *Streptomyces* 85E strain (28 ± 0.35 mm bald phenotype at 100 μg/disc; MIC = 12.5 μg/ disc) while, Chloroform extract displayed maximum antidiabetic activity (α-amylase inhibition of 21.61 ± 1.53% at 200 μg/ml concentration). The highest antileishmanial potential was found in Ethyl acetate-Acetone extract (12.91 ± 0.02% at 100 μg/ml concentration), while, *Q. dilatata* extracts also showed a moderate antifungal activity.

**Conclusion:**

This study proposes that multiple-solvent system is a crucial variable to elucidate pharmacological potential of *Q. dilatata* and the results of the present findings prospects its potential as a resource for the discovery of novel anticancer, antidiabetic, antileishmanial and antioxidant agents.

## Background

Bioactive phytoconstituents include an array of compounds (e.g., tannins, lignans, coumarins, quinones, stilbenes, xanthones, phenolic acids, flavones, flavonols, catechins, anthocyanins, and proanthocyanins) that could delay or deter the inception of diseases such as cancer and diabetes [1]. They have also contributed to the pharmacopeia of the world for the provision of new and effective agents against infectious diseases such as leishmaniasis [2]. Mounting the credibility of traditional medicinal plants requires consolidation of scientific data to fill the research based evidence gaps. Therefore, the present study is designed to appraise the antioxidant, cytotoxic, kinases inhibitory, antileishmanial and antidiabetic properties of a folklore medicinal plant; *Quercus dilatata*.

*Quercus dilatata* Lindl. ex Royle (Synonym; *Quercus floribunda* Lindl. Camus), known commonly as Holly Oak and locally as Bunj or Barungi, belongs to family Fagaceae. The genus *Quercus* encompassing around 400 species is distributed in America, temperate Europe, Asia and sub-tropical Africa. In Pakistan this evergreen tree is abundant in the Himaliyan mountains specially in Dir, Chitral, Swat, Hazara, Tirah, Kurram Agency, Murree hills and Azad Kashmir [3]. Powdered form of its fruits are utilized for the eradication of gonorrhoea and urinary tract infections in district Swat [4]. Leaves and seeds are utilized against sore mouth and throat in Lawat district [5]. Seeds are also believed to be astringent, diuretic and are employed in diarrhoea, indigestion and asthma in Poonch Valley [6].

Extraction efficiency and bioactivity of herbal extracts can be optimized by varying extraction solvent polarity [7]. In this study, multiple mono and binary solvent systems of escalating polarities were applied to find out most proficient system for the extraction of antioxidant, cytotoxic, protein kinase inhibitory, antidiabetic and/or antimicrobial potential of *Q. dilatata*. To the best of our knowledge, protein kinase inhibitory, antileishmanial, antidiabetic and cytotoxic (against THP-1 and Hep G2 human cancer cell lines) activities of *Q. dilatata* extracts are being reported for the first time here.

## Methods

### Solvents and reagents

Solvents (n-Hexane, chloroform, acetone, ethyl acetate, methanol, ethanol and DMSO), gallic acid, quercetin, aluminium chloride (AlCl_3_), potassium acetate, 2,2-diphenyl-1-picrylhydrazyl (DPPH), ascorbic acid, sulfuric acid (H_2_SO_4_), ammonium molybdate, monosodium dihydrogen phosphate (NaH_2_PO_4_), trichloroacetic acid (TCA), potassium ferricyanide, ferric chloride (FeCl_3_), standard antibiotics (cefixime, ciprofloxacin), standard antifungal (clotrimazole), trypton soy broth (TSB), α-amylase enzyme (from *Bacillus subtilis*), acarbose, phosphate buffer (PB), Folin–Ciocalteu reagent, RPMI-1640 medium, Medium 119, DMEM and sea salt were purchased from Sigma (Sigma-Aldrich Germany). Sabouraud dextrose agar (SDA) was purchased from Oxoid England; Tween-20 from Merck-Schuchardt, USA. Medium ISP4 was prepared in lab while doxorubicin was purchased from Merck (Darmstadt, Germany).

### Collection and extraction

Aerial parts of *Q. dilatata* were collected during September 2013 from Murree hills, Pakistan and identified by Prof. Dr. Rizwana Aleem Qureshi, Department of Plant Sciences, Faculty of Biological Sciences, Quaid-i-Azam University Islamabad, Pakistan. Voucher specimen (PHM-490) was archived in the herbarium of medicinal plants, Department of Pharmacy, Quaid-i-Azam University, Islamabad. The plant was thoroughly washed under running water and shade dried with active ventilation at ambient temperature for three weeks. The dried plant material was pulverised to fine powder and stored in air-tight containers till further use. The powdered plant (40 g) was subjected to sonication aided maceration for 24 h at room temperature using analytical grade solvents (400 ml) in 1000 ml Erlenmeyer flask. The extracts were concentrated by vacuum evaporation in rotary evaporator (Buchi, Switzerland) and dried in vacuum oven (Yamato, Japan) at 45 °C to obtain final crude extract. The experiment was run in triplicate. The solvents included n-Hexane (NH), Chloroform (C), Acetone (A), Ethyl acetate + Acetone (EtA), Ethyl acetate (Et), Chloroform + Ethanol (CE), Chloroform + Methanol (CM), Ethanol + Ethyl acetate (EEt), Methanol + Ethyl acetate (MEt), Ethanol (E), Distilled water + Acetone (DA), Methanol (M), Distilled water + Methanol (DM) and Distilled water (D). A ratio of 1:1 was used for the preparation of binary solvent systems. The extracts were stored at −30 °C for further testing.

### Extract recovery

The dried extracts were weighed to calculate % recovery of crude extracts by the following formula.

$$ \mathrm{Extract}\ \mathrm{recovery}\ \left(\%\mathrm{w}/\mathrm{w}\right)=\left(\mathrm{A}/40\right)\times 100 $$

Where; A = weight of crude extract obtained after drying.

### Phytochemical analysis

Stock solution of each crude extract in DMSO (4 mg/ml) was prepared for phytochemical analysis.

### Determination of total phenolic content (TPC)

Folin-Ciocalteu (FC) reagent method was used to estimate total phenolic content [8, 9]. An aliquot of 20 μl from stock solution and 90 μl of FC reagent in 96 well plate was incubated for 5 min at room temperature followed by the addition of 90 μl of sodium carbonate solution. Absorbance of the assay plate was recorded at 630 nm using microplate reader (Biotech USA, microplate reader Elx 800). A calibration curve (y = 0.0136× + 0.0845, R^2^ = 0.9861) was obtained under the same operating conditions using gallic acid (6.25–50 μg/ml) as a positive standard. The assay was performed in triplicate and the results are expressed as microgram gallic acid equivalent per milligram dry weight (μg GAE/mg DW).

### Determination of total flavonoid content (TFC)

Total flavonoid content was estimated by aluminium chloride colorimetric method with minor modifications [10, 11]. Briefly 20 μl from each test sample stock solution, 10 μl of aluminium chloride (10% *w*/*v* in H_2_O), 10 μl of 1.0 M potassium acetate and 160 μl of distilled water were added in 96 well plate which was incubated at room temperature for 30 min. The absorbance of the plate was measured at 415 nm using microplate reader. A calibration curve (y = 0.0268× + 0.00764, R^2^ = 0.9851) of quercetin was drawn at final concentrations of 2.5, 5, 10, 20, 40 μg/ml and the resultant flavonoid content is expressed in microgram equivalents of quercetin per milligram dry weight (μg QE/mg DW).

### RP-HPLC quantitative analysis

High performance liquid chromatography was performed according to previously described procedure [12, 13]. Dried extracts (0.5 g) were dissolved in methanol (62.5%) and 6 M HCl solution. After purging nitrogen for few sec, samples were refluxed for 2 h. Filtered extracts were adjusted to 100 ml (with methanol) and re-filtered through 0.45 μm membrane filter (Millex-HV) before injecting into HPLC. The HPLC system (Shimadzu LC-20AT) was equipped with auto-sampler (SIL-20A), column oven (CTO-20A), and diode array detector (SPD-M20A). Analytical column- Nucleosil C18, 5 μm 100 A° (250 × 4.60 mm, Phenomenex) coupled with a guard column (KJO-4282, Phenomenex) was used. Mobile phase was composed of 1% acetic acid solution and 70% methanol. Gradient program by Araruna et al. [14] was used with a flow rate of 0.8 ml/min. Phenolic compounds were identified by comparing retention time and UV–vis spectra of chromatographic peaks with that of authentic reference standards at 280 nm.

### Biological evaluation

#### Antioxidant assays

##### Free radical scavenging activity (FRSA)

The FRSA of the crude extracts was evaluated by monitoring their capability to quench the stable 2, 2-diphenyl-1-picrylhydrazyl (DPPH) free radical [8, 15]. Briefly, 20 μl of four different dilutions of each test sample to have final concentrations of 200, 66.66, 22.22 and 7.41 μg/ml, were mixed with 180 μl of DPPH solution (9.2 mg/100 ml methanol) in 96 well plate. After incubating the plate for 30 min at 37 °C, absorbance was recorded at 517 nm. Percent free radical scavenging activity (%FRSA) was calculated by using the formula:

$$ \%\mathrm{FRSA}=\left(1\hbox{--} {\mathrm{Ab}}_{\mathrm{s}}/{\mathrm{Ab}}_{\mathrm{c}}\right)\times 100 $$

Where Ab_s_ is the absorbance of test sample, whereas Ab_c_ is the absorbance of negative control containing the DMSO instead of sample. Ascorbic acid was used as positive control and the assay was performed in triplicate. Afterwards IC_50_ of samples with significant radical scavenging efficiency (>50%) was also calculated.

##### Total antioxidant capacity (TAC)

Phosphomolybdenum based colorimetric assay was employed to determine total antioxidant capacity and is expressed as the number of microgram equivalents of ascorbic acid per milligram of dry plant weight (μg AAE/mg DW) [10, 16]. An aliquot of 0.1 ml of each test extract (4 mg/ml DMSO) and positive control (ascorbic acid, 4 mg/ml DMSO) was mixed with 0.9 ml of reagent (0.6 M sulphuric acid, 28 mM sodium phosphate and 4 mM ammonium molybdate solution in H_2_O). Blank contained 0.9 ml of reagent solution and 0.1 ml of DMSO without extract. All tubes were kept in water bath for 90 min at 95 °C and then cooled to room temperature from which 200 μl of each sample was transferred to 96 well plate and the absorbance of each sample was measured at 630 nm using microplate reader (Biotech USA, microplate reader Elx 800). A calibration curve (y = 0.0212× + 0.0926, R^2^ = 0.9913) of ascorbic acid was drawn at final concentrations of 100, 50, 25, 12.5, 6.25, 3.12 μg/ml and the experiment was performed in triplicate.

##### Total reducing power (TRP)

Standard potassium ferricyanide colorimetric assay was performed to estimate the reducing power of different solvent extracts [10, 17]. An aliquot of 200 μl of test extracts (4 mg/ml DMSO) was mixed with 400 μl of each phosphate buffer (0.2 mol/l, pH 6.6) and potassium ferricyanide (1% *w*/*v* in H_2_O). The mixture was incubated for 20 min at 50 °C followed by addition of 400 μl of trichloroacetic acid (10% *w*/*v* in H_2_O) and centrifuged at 3000 rpm at room temperature for 10 min. The upper layer of solution (500 μl) was mixed with distilled water (500 μl) and 100 μl of FeCl_3_ (0.1% *w*/*v* in H_2_O). From this mixture, 200 μl was transferred to 96 well plate and the absorbance of the reaction mixture was measured at 630 nm. Blank was prepared by adding 200 μl of DMSO to the aforesaid reaction mixture instead of extract. A calibration curve (y = 0.038× + 0.7484, R^2^ = 0.9967) of ascorbic acid was obtained at final concentrations of 100, 50, 25, 12.5, 6.25, 3.12 μg/ml and the resultant reducing power of each sample is expressed as μg AAE/mg DW. The assay was performed in triplicate.

### Cytotoxicity assays

#### Brine shrimp lethality assay

A 24 h lethality test was performed in a 96 well plate against brine shrimp (*Artemia salina*) larvae as described previously [8]. Eggs of *A. salina* (Ocean star, USA) were incubated for 24–48 h hatching period under light and warmth (30–32 °C) in simulated sea water (38 g/l supplemented with 6 mg/l dried yeast) in a specially designed two-compartment plastic tray. The mature phototropic nauplii (10) were then harvested with the help of Pasteur pipette and transferred to each well of plate. Corresponding volume of each extract containing ≤1% DMSO in sea water at final concentrations of 200, 100, 50 and 25 μg/ml was transferred to the wells containing sea water and shrimp larvae. The final volume in each well was kept 300 μl. Positive and negative control wells included serial concentrations of doxorubicin and 1% DMSO in sea water respectively. After 24 h incubation, live shrimps were counted and the percentage of deaths was determined. Median lethal concentration (LC_50_) was calculated using table curve 2D v5.01 software. The experiment was run in triplicate.

#### Cytotoxicity against THP-1 human leukemia cell line

The in vitro cytotoxicity evaluation of extracts against human leukemia (THP-1) cell line (ATCC # TIB-202) was performed using standard protocol [18]. Concisely, leukemia cells were grown in complete growth medium [RPMI-1640 buffered with 2.2 g/l NaHCO_3_ and supplemented with 10% *v*/v heat inactivated foetal bovine serum (HIFBS); pH 7.4] in a humidified carbon dioxide incubator (37 °C, 5% CO_2_). 10 μl of test sample containing 1% DMSO in PBS was added in wells of microtitre plate to have a final concentration of 20 μg/ml. Subsequently, an aliquot of 190 μl of THP-1 cells (seeding density of 1 × 10^4^ cells per ml) was transferred to each well. The culture was incubated at 37 °C for 72 h in humidified CO_2_ (5%) incubator (Panasonic, Japan MCO-18 AC-PE). Serial concentrations of fluorouracil and vincristine were employed as positive controls whereas 1% DMSO in PBS served as negative control. Afterwards, 20 μl of pre-filter sterilized MTT solution (4 mg/ml in distilled H_2_O) was added and plates were again incubated at 37 °C for 4 h in humidified CO_2_ (5%) incubator. After incubation supernatant was removed carefully by multi-channel pipette without disturbing coloured formazan sediments, which were equivalent to amount of live cells. To dissolve the formazan sediments 100 μl of DMSO was added in each well, the plate was kept aside for 1 h to ensure full dissolution and the absorbance was measured at 540 nm using microplate reader. Samples showing more than 50% cell mortality at 20 μg/ml were further analysed at lower concentrations i.e. 10, 5, 2.5 and 1.25 μg/ml and the assay was performed in triplicate. IC_50_ was calculated by using table curve 2D v5.01 software.

#### Cytotoxicity against Hep G2 cell line

The cytotoxic potential of extracts towards Hep G2 cancer cell line (RBRC-RCB1648) was determined by using SRB colorimetric assay as described previously [19]. Briefly, Hep G2 cells were grown in Dulbecco’s Modified Eagle Medium (DMEM) supplemented with 10% *v*/v FBS, 100 IU/ml penicillin G sodium, 100 μg/ml streptomycin sulphate and 0.25 μg/ml amphotericin B. The plate was then incubated in humidified atmosphere at 37 °C and 5% CO_2_ for 72 h to obtain a confluence of approximately 60–70%. Old medium was replaced with fresh medium and the cells were incubated for another 24 h after which they were trypsonised and diluted to get an assay density of 1 × 10^5^ cells/ml. An aliquot of 180 μl from aforementioned culture was then transferred to each well of 96 well plate having 20 μl of test samples (containing 1% DMSO in PBS) to have final concentration of 20 μg/ml. Doxorubicin (20–0.08 μg/ml) and 1% *v*/v DMSO in PBS instead of test sample were employed as positive and negative controls respectively. The culture plate was then incubated at 37 °C for 72 h in CO_2_ incubator. The incubation was stopped with the addition of 50 μl of cold 20% *w*/*v* TCA for 1 h at 4 °C for cell fixation. The fixed cells were washed 4 times with tap water, air dried and stained with 50 μl of 0.057% *w*/*v* SRB in 1% *w*/*v* acetic acid for 30 min at room temperature. Wells were then washed 4 times with 1% *v*/v acetic acid and the plates were dried overnight. Bound dye was solubilized in 200 μl 10 mM Tris base, pH 10, for 1 h. Optical density was measured on a micro-plate reader (Biotech USA, microplate reader Elx 800) at 515 nm and percent survival was determined. In each case, a zero-day control was performed by adding an equivalent number of cells to sixteen wells, incubating at 37 °C for 1 h and processing as described above. Percent of cell growth inhibition was calculated using the formula:

$$ \%\mathrm{Inhibition}=100\hbox{--} \left[\left({OD}_{\mathrm{cells}+\mathrm{samples}}\hbox{--} {OD}_{\mathrm{day}\ 0}\right)/\left({OD}_{\mathrm{cells}+1\%\mathrm{DMSO}}\hbox{--} {OD}_{\mathrm{day}0}\right)\times 100\ \right] $$

#### Cytotoxicity against isolated lymphocytes

Lymphocytes were isolated using formerly described procedure with slight modifications [20]. Informed consent was taken from volunteers and protocol was followed according to international ethical guidelines after approval from the ethical committee of the Quaid-i-Azam University (IRB-QAU-116). A volume of 3 mL of blood was collected from a healthy donor by venipuncture and diluted (1:1) with PBS. It was layered over 2 mL Histopaque-1077 and centrifuged at 800×g for 20 min. The buffy coat was aspirated into 5 mL of PBS and centrifuged at 350 rpm for 4 min to pellet the lymphocytes. The pellet was suspended in 1 mL of RPMI-1640 and cell density was adjusted to get 1 × 10^5^ cells/ml. For cytotoxicity determination, 20 μl of samples (20 μg/ml) or vincristine or 1% DMSO in PBS and 180 μl of lymphocyte suspension were incubated in 96-well plate at 37 °C for 24 h in humidified 5% carbondioxide incubator (Panasonic, Japan MCO-18 AC-PE). Phytohaemagglutinin (PHA) was added in medium to stimulate lymphocyte growth. Afterwards, MTT assay was performed as described above.

#### Protein kinase inhibition assay

The protein kinase inhibition assay was performed by observing hyphae formation in purified isolates of *Streptomyces* 85E strain [21]. Microbial lawn was formed by spreading spores and mycelia fragments (100 μl) of refreshed culture of Streptomyces on plates containing minimal ISP4 medium under sterile conditions. An aliquot of 5 μl of each extract (20 mg/ml of DMSO) was loaded onto sterile 6 mm filter paper discs. The impregnated sterile discs each having 100 μg/disc of extract were placed on the surface of the plates seeded with *Streptomyces* 85E. Surfactin (5 μl of 4 mg/ml of DMSO) and DMSO impregnated discs served as positive and negative controls respectively. The plates were incubated for 72–96 h at 30 °C (time required for hyphae formation in *Streptomyces* 85E). Results were noted for the presence of bald or clear zone of inhibition around samples and control discs.

#### Antileishmanial assay

The in vitro antileishmanial evaluation of test extracts was carried out by employing MTT colorimetric assay as described previously [22]. A 6–7 days incubated culture of *Leishmania tropica* kwh 23 promastigotes was used. Concisely, parasites were grown in Medium 199 supplemented with 10% foetal bovine serum (FBS), 100 μg/ml streptomycin sulphate and 100 IU/ml penicillin G at 24 °C. An aliquot of 180 μl of promastigote culture at a pre-adjusted seeding density of 1 × 10^6^ promastigotes/ml was transferred to each well of 96 well plate having 20 μl of test samples (containing ≤1% DMSO in PBS) at concentration of 100 μg/ml. Amphotericin B (0.33–0.004 μg/ml) and 1% DMSO in PBS instead of test sample were employed as positive and negative controls respectively. The culture plate was then incubated at 24 °C for 72 h after which 20 μl of pre-filter sterilized MTT solution (4 mg/ml in distilled H_2_O) was added and plates were again incubated at 24 °C for 4 h. After incubation supernatant was removed carefully without disturbing coloured formazan sediments. To dissolve the formazan sediments, 100 μl of DMSO was added in each well, the plate was kept aside for 1 h to ensure full dissolution and the absorbance was measured at 540 nm using microplate reader. Samples showing more than 50% cell mortality at 100 μg/ml were further analysed at lower concentrations i.e. 33.3, 11.1, 3.7 and 1.23 μg/ml. IC_50_ was calculated by using table curve 2D v5.01 software.

#### α-Amylase inhibition assay

Antidiabetic potential of test extracts was determined by α-amylase inhibition assay following the standard protocol with minor modification [23]. The reaction mixture containing 15 μl phosphate buffer (pH 6.8), 25 μl α-amylase enzyme (0.14 U/ml), 10 μl sample (4 mg/ml DMSO) and 40 μl starch solution (2 mg/ml in potassium phosphate buffer) was incubated at 50 °C for 30 min in 96 well plate followed by addition of 20 μl of 1 M HCl to stop the reaction. Afterwards 90 μl of iodine reagent (5 mM iodine, 5 mM potassium iodide) was added to each well. Negative control was prepared without plant extracts whereas blank was prepared without extracts and amylase enzyme; each being replaced by equal quantities of buffer. Acarbose (250 μM) was used as positive control. Absorbance of reaction plate after incubation was measured at 540 nm. Activity was expressed as percent α-amylase inhibition/mg dry extract and calculated by the following equation:

$$ \%\upalpha \hbox{--} \mathrm{amylase}\ \mathrm{inhibition}=\left(\mathrm{Os}\hbox{--} \mathrm{On}\right)/\left(Ob\hbox{--} \mathrm{On}\right)\times 100 $$

where *On* = Absorbance of negative control, *Os* = Absorbance of sample and *Ob* = Absorbance of blank well.

#### Antifungal assay

The antifungal activity of test extracts was evaluated by agar disc diffusion method performed in triplicate [24]. The spores of test fungal strains [*Aspergillus fumigatus* **(**FCBP**-** 66)**,** *Mucor* species (FCBP-0300), *Aspergillus niger* (FCBP-0198) and *Aspergillus flavus* (FCBP-0064)] were harvested in 0.02% *v*/v Tween 20 solution in H_2_O and their turbidity was adjusted according to McFarland 0.5 turbidity standard. Then 100 μl of each harvested fungal strain was swabbed on plates containing Sabouraud Dextrose agar. Sterile filter paper discs impregnated with 5 μl of test extracts (20 mg/ml of DMSO), positive control (Clotrimazole, 4 mg/ml of DMSO) and negative control (DMSO) were placed on seeded plates and incubated for 24–48 h at 28 °C. Afterwards, the average diameter (mm) of growth inhibition zone around the samples as well as control treated discs was measured and recorded.

### Statistical analysis

Data were expressed as mean ± SD. The results obtained for phytochemical and cytotoxic assays were analysed statistically by one-way analysis of variance (ANOVA using the statistical package PASW Statistics 18).

## Results and discussion

### Effect of extraction solvent on the extract yields

**Fig. 1:**
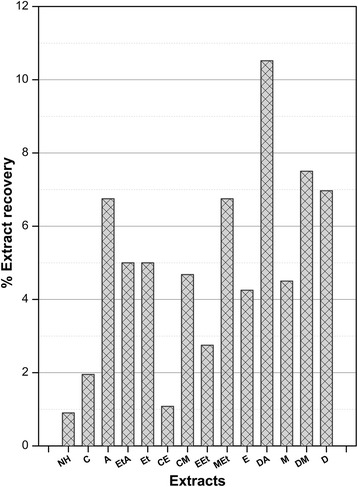
Percent extract recovery of *Q. dilatata* using mono and binary (1:1) solvents for extraction. Values are presented as mean ± standard error from triplicate investigation. NH: n-hexane, C: Chloroform, A: Acetone, EtA: Ethyl acetate + Acetone, Et: Ethyl acetate, CE: Ethanol + Chloroform, CM: Chloroform + Methanol, EEt: Ethanol + Ethyl acetate, MEt: Methanol + Ethyl acetate, E: Ethanol, DA: Distilled water + Acetone, M: Methanol, DM: Distilled water + Methanol, D: Distilled water

The percentage recovery of extracts prepared by employing a total of 14 mono and binary solvent systems of escalating polarity has been summarized in Fig. 1. Maximum amount of extract was recovered when DA was used as the extraction solvent with an extract yield of 10.52% *w*/w followed by D (6.97% *w*/w) and A (6.75% *w*/w) extracts respectively. On the other hand, it was least (0.9% *w*/w) in case of NH extract. An extraction procedure with superlative efficiency with respect to time/yield ratio is fundamental to accurately quantify the phytoconstituents [25]. This in turn also affects the biological evaluation of extracts. We observed a decreasing trend in extract yield along with decreasing polarity of extraction solvent. Thus, the use of sonication aided maceration with wide ranging solvent polarities in our study is a critical parameter in extraction yield optimization and its correlation with biological activities. This observation is in agreement with the previous report where effects of different extraction solvents, used in two extraction methods, on the total polyphenol contents of *Q. coccifera* fruits were studied and it was found that solvents with different polarities had significant effects on antioxidant activity [26]. The differences in the extract yields can be attributed to variable solubility of phytoconstituents based on their varied chemical composition in the plant [22].

### Phytochemical analysis

In vitro phytochemical and antioxidant assays were performed by drawing a dose–response (calibration) curve of the reference compounds at multiple doses. The calculation of the sample was made in triplicate by using the equation drawn from the above curve.

### TPC

**Fig. 2:**
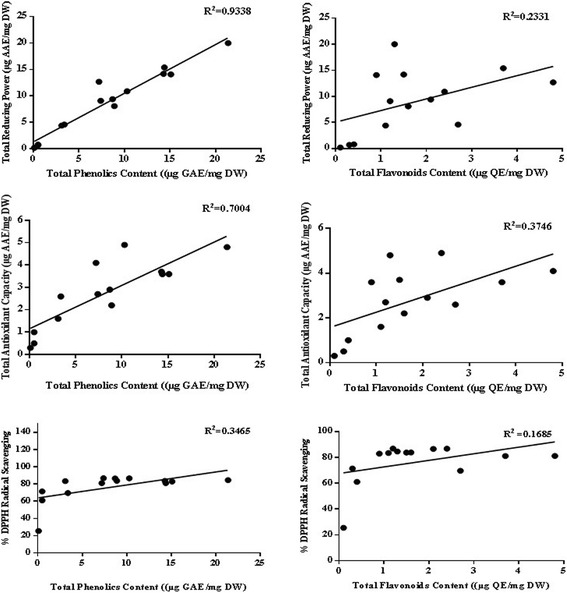
Correlation of total phenolic and flavonoid content with antioxidant potential determining assays

**Fig. 3:**
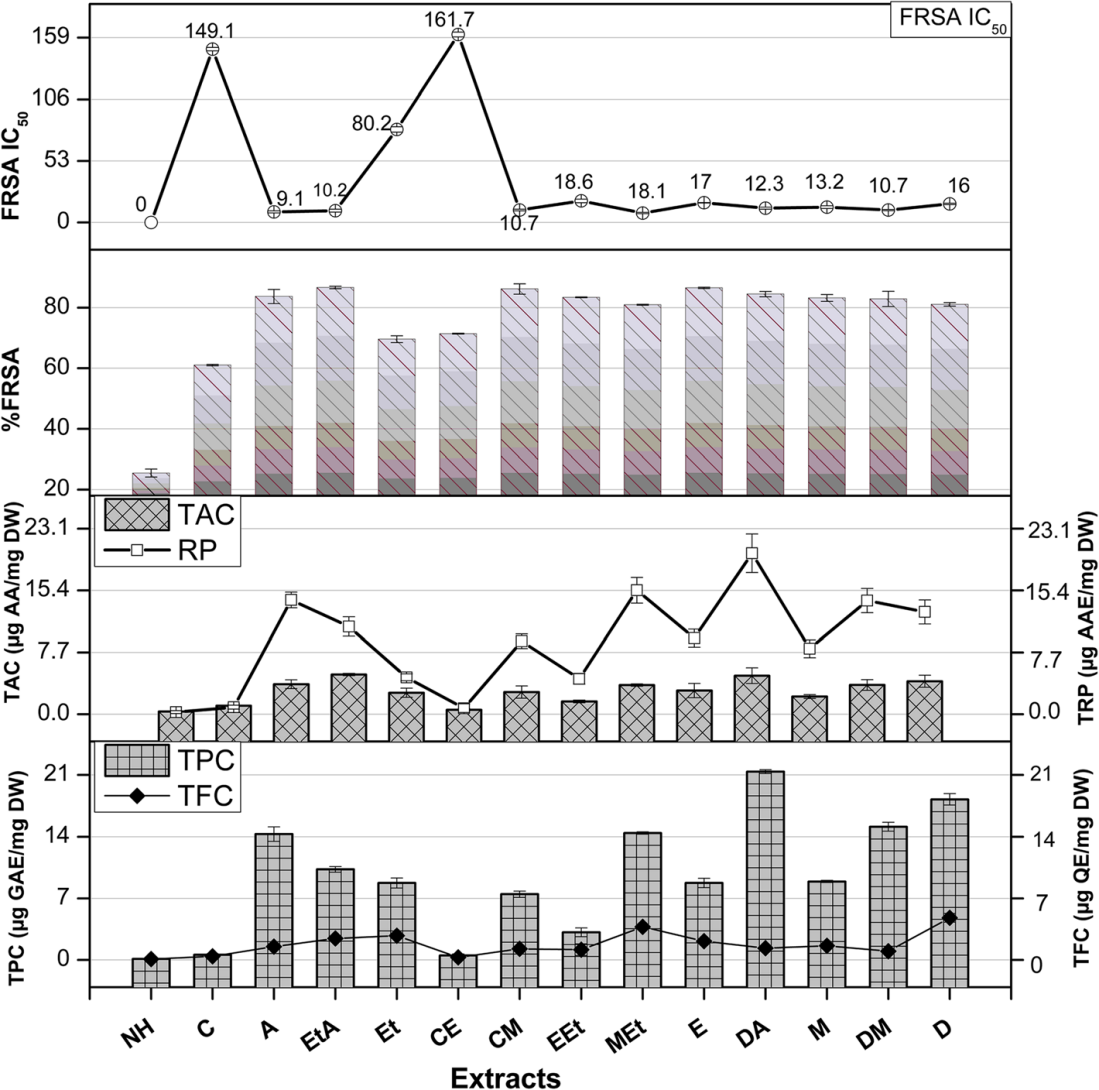
TPC (Total phenolic content μg GAE/mg DW), TFC (Total flavonoid content μg QE/mg DW), TAC (Total antioxidant capacity μg AAE/mg DW), TRP (Total reducing power μg AAE/mg DW) and %FRSA (radical scavenging activity) of *Q. dilatata* in different solvents*.* Values are presented as mean ± Standard deviation from triplicate investigation

The total gallic acid equivalent phenols in *Q. dilatata* extracts ranged from 21.37 ± 0.21 to 0.12 ± 0.01 μg GAE/mg DW with the highest content quantified in the DA extract. The phenolic content decreased in accordance with the following order; DA > D > DM > MEt > A > EtA > M > Et = E > CM > EEt > C > CE > NH (Fig. 3). Since, the polarities of polyphenols range from polar to non-polar, thus a wide range of solvents is required for their extraction. It has been observed that polar binary solvent systems are more efficient than mono solvent systems in the extraction of polyphenolic compounds which is in agreement with the results of previous study [27]. Thus polarity plays a key role in increasing solubility of phenols. A significant relationship between antioxidant capacity, reducing power and total phenolic content was found in this exploration, indicating that phenolic compounds are the major contributors to the antioxidant properties of this plant (Fig. 2). High levels of polyphenols are also reported in other species of *Quercus* such as *Q. robur, Q. coccifera* [26, 28]. The HPLC-MS data of phenolic fraction of cork from *Q. suber* identified 15 phenolic components, with ellagic acid, followed by gallic and protocatechuic acids as the most abundant compounds [29]. The plant phenolics possess diverse biological activities such as anthelmintic [30], antifungal [31], antibacterial [32], antitumor [33], antiviral [34], antioxidant [35] and hepatoprotective [36]. The antioxidant properties have an important role in combating oxidative stress, cytotoxicity and cell death by scavenging free radicals or chelating trace elements thereby strengthening the antioxidant defences [35]. Therefore, the presence of significant amounts of phenolic content in *Q. dilatata* proposes it a natural hub of the aforementioned pharmacological attributes.

### TFC

The total flavonoid content of *Q. dilatata* aerial parts in terms of μg QE/mg DW are presented in Fig. 3. Among all the extracts, highest flavonoid content of 4.78 ± 0.51 μg QE/mg DW was recorded in aqueous extract followed by MEt extract (3.76 ± 0.16 μg QE/mg DW). Lowest content was observed when NH was used alone with the value of 0.09 ± 0.01 μg QE/mg DW. The flavonoid content decreased with polarity in the following order; D > MEt > Et > EtA > E > M > A > DA > EEt > CM > DM > C > CE > NH. The genus *Quercus* is reported to be rich in flavonoid polyphenols as Brossa et al. [37] established that major constituents in *Q. ilex* leaves are flavanols and flavonols while *Q. petraea* and *Q. robur* are also found to be rich in flavonoids [38]. The compounds such as flavonoids, which hold hydroxyls groups, are responsible for the free radical scavenging activity in the plants and act through scavenging or chelating process [39]. Thus, the current flavonoid determination in polar extracts of *Q. dilatata* suggests it to be a valuable natural antioxidant.

### RP-HPLC

**Fig. 4:**
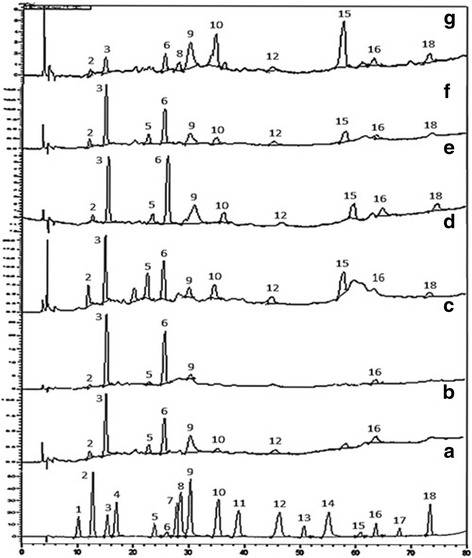
RP-HPLC chromatograms of standard compounds (**a**) and DA (**b**), D (**c**), MEt (**d**), CM (e), M (**f**) and Et (**g**) extracts of *Quercus dilatata* showing phenolic compounds Hydroquinone (1), Pyrocatechol (2), Gallic acid (3), Resorcinol (4), Catechin (5), Chlorogenic acid (6), Caffeic acid (7), Vanillic acid (8), p-Coumaric acid (9), Ferulic acid (10), Sinapic acid (11), Coumarin (12), Salicylic acid (13), Trans cinnamic acid (14), Rutin (15), Quercetin (16), Ellagic acid (17) and Kaempferol (18). Values represented by bold letters are maximum phenolic contents detected in test samples

Quantitative analysis of polyphenols as well as chromatographic fingerprinting of bioactive samples was done by RP-HPLC profiling (using 18 standards), after comparing their chromatographs with those of standards (Fig. 4). Quantitative determination of 11 detected polyphenols has been presented in Table 1. Maximum contents of gallic acid, chlorogenic acid and p-coumaric acid were expressed in DA extract (5.282, 15.336 and 1.026 μg/mg extract respectively), while highest contents of pyrocatechol, catechin, coumarin and quercetin were observed in MEt extract (0.361, 2.291, 0.368 and 0.969 μg/mg extract respectively). In addition, maximum contents of vanillic acid, ferulic acid, rutin and kaempferol were observed in Et extract (1.271, 0.701, 1.181 and 0.291 μg/mg extract, respectively). Various solvents systems have been employed for the isolation of polyphenols. Their extraction efficiency is mainly effected by the choice of solvents and method of extraction [40]. Our results clearly indicate an increase in levels of detected polyphenols with ascending polarity of extraction solvents. Highest amounts of phenolic compounds were detected in DA mixture, which is supported by previous report in which aqueous acetone mixture (70%) was found to be most effective for isolation of maximum amounts of condensed tannins from peas [41]. In terms of bioactivities, it is evident from the results that highest antioxidant potential shown by DA and MEt extracts might be attributed to the presence of hydroxycinnamic acids (chlorogenic acid, coumarin, p-coumarin), hydroxybenzoic acids (gallic acid) as well as flavonols (quercetin) and flavan-3- ols (catechin). Previous reports also support this hypothesis in which phenolics have been considered as strong candidates with potential antioxidant activity [42, 43]. In addition to their antioxidant properties, these phenolic compounds have also shown chemopreventive and cytotoxic effects both in in vitro and in vivo models. Anthocyanins, kaempferol, quercetin, esters of coumaric acid and ellagic acid were found to be inhibitors of human oral (KB, CAL-27), breast (MCF-7), colon (HT-29, HCT-116), and prostate (LNCaP, DU-145) tumor cell lines in a dose-dependent manner [44]. Similarly, polyphenol constituents such as epigallocatechin-gallate (EGCG) and theaflavin have been demonstrated as potent anticancer agents in various animal models [45]. These studies suggest the possible role of polyphenols for the induction of cytotoxic potential of *Q. dilatata* extracts in the present study. Chromatograms of standards as well as phenols detected in the samples have been presented in Fig. 4.

**Table 1:** Phenolic composition (μg/mg extract) of six different *Quercus dilatata* extracts

Standards	Retention time (min)	Samples					
		Et	CM	MEt	DA	M	D
Hydroquinone	10.07	--	--	--	--	--	--
Pyrocatechol	12.06	0.049	0.013	0.361	0.232	0.111	0.017
Gallic acid	15.24	0.534	0.499	2.822	5.282	2.112	1.382
Resorcinol	17.48	--	--	--	--	--	--
Catechin	23.61	--	0.150	2.291	1.380	0.808	0.095
Chlorogenic acid	25.80	1.009	2.699	9.478	15.336	7.122	5.162
Caffeic acid	27.51	--	--	--	--	--	--
Vanillic acid	28.21	1.271	--	--	--	--	--
p-Coumaric acid	30.16	0.534	0.146	0.232	1.026	0.339	0.036
Ferulic acid	34.87	0.701	0.046	0.461	0.173	0.192	--
Sinapinic acid	38.48	--	--	--	--	--	--
Coumarin	44.85	0.105	0.105	0.368	--	0.149	--
Salicylic acid	50.05	--	--	--	--	--	--
Trans cinnamic acid	54.44	--	--	--	--	--	--
Rutin	58.79	1.181	0.094	--	0.245	0.407	--
Quercetin	63.13	0.164	0.060	0.969	0.616	0.085	0.059
Ellagic acid	67.35	--	--	--	--	--	--
Kaempferol	73.06	0.291	0.069	0.284	--	0.129	--

- Not detected

### Biological evaluation

#### Antioxidant assays

##### FRSA

The percent free radical scavenging activity (% FRSA) of test samples, evaluated by measuring the discoloration of DPPH solution is shown in Fig. 3. The assay protocol relies on the conversion of stable purple coloured DPPH radical to its yellow-coloured diphenyl picryl hydrazine molecule by accepting electron or hydrogen radical from the donor antioxidant. The DPPH molecule is regarded as a stable free radical owing to the presence of a delocalized spare electron over the entire molecule that gives a characteristic absorption band at 517 nm [46]. Highest free radical scavenging efficiency was exhibited by MEt extract (IC_50_ = 8.1 ± 0.5 μg/ml) followed by A extract (IC_50_ = 9.1 ± 0.89 μg/ml). The scavenging effect of plant extracts on the DPPH radical decreased in the following order: MEt > A > EtA > CM > DM > DA > M > D > E > EEt > Et > C > CE > NH. The radical scavenging efficiency of MEt, A, EtA, CM, DM and DA extracts is comparable, but the high extraction yields obtained by DA extract opens good perspectives for the exploitation of this extract in nutraceutical applications. The current scavenging results also support the previous findings where the polar ethyl acetate fraction of *Q. dilatata* exhibited the highest antiradical potential (IC_50_ = 38.02 μg/ml) while the least quenching effect was manifested by the non-polar n-hexane fraction [3]. Previously, the FRSA of *Q. subar and Q. coccifera* extracts, evaluated using this assay was found to be considerably higher than BHT and comparable to that of BHA (3-t-butyl-4-hydroxyanisole), quercetin and ascorbic acid [29, 47].

##### TAC

The total antioxidant capacity (TAC) of various solvent soluble extracts of *Q. dilatata* is summarized in Fig. 3. The assay is based on antioxidant mediated reduction of Mo (VI) to Mo (V) resulting in the formation of green coloured phosphate/Mo (V) complex [22]. The total antioxidant capacity of the extracts was found to decrease in order of DA extract (4.81 ± 0.98 μg AAE/mg DW) > D > EtA > A > DM > MEt > E > CM > Et > M > EEt > C > CE > NH extract with the value of 0.34 ± 0.10 μg AAE/mg DW. The presence of a significant correlation between TAC and TPC (R^2^ = 0.7004) and a non-significant correlation between TAC and TFC (R^2^ = 0.3746) suggests that phenols other than flavonoids are the major contributors towards the antioxidant activity of *Q. dilatata* crude extracts (Fig. 2). Previously the total antioxidant activity of methanol extract was reported as 64 mg equivalent of BHT/g of *Q. infectoria* nutgalls which is quite high in comparison to the antioxidant capacity of the methanolic extract of *Q. dilatata* aerial parts (2.20 ± 0.25 μg AAE/mg DW) as determined in the current analysis [39]. However, overall yield of aerial parts per plant is also much higher than the nutgalls. This still buttress the potential of this plant as a source of natural antioxidants.

##### TRP

Figure 3 shows the reductive power of various solvent extracts of *Q. dilatata*. It was observed that the maximum extraction efficiency in terms of highest reducing power was achieved in the DA extract (20.03 ± 2.4 μg AAE/mg DW). The total reducing capacity of test extracts was found to decrease in order of DA > MEt > A > DM > D > EtA > E > CM > M > Et > EEt > C > CE > NH. A significant relationship between TRP and TPC (R^2^ = 0.9338) while a non-significant correlation between TRP and TFC (R^2^ = 0.2331) is observed in this study (Fig. 2). Therefore, it is suggested that phenolic compounds might be the major contributors to the reducing properties of this plant. The various antioxidant potential assays of medicinal plants used in diet therapy during postpartum healthcare in Rajasthan, India revealed that among all the plants tested, the *Q. infectoria* extract possessed highest FRSA and reducing potential of 90.20% of DPPH inhibition and 1115 mM Fe^+2^/g respectively [48]. The reducing properties are generally associated with the presence of reductones which have been allied to the antioxidant action through breakage of the free radical chain by donating a hydrogen atom. Consequently, a direct correlation have been observed between the antioxidant capacity and reducing power of certain plant extracts which is in agreement with the results of our study [49].

### Cytotoxicity assessment

#### Brine shrimp lethality assay

Cytotoxicity prospective of the plant was tested against brine shrimp larvae to establish its bioactivity profile. The degree of lethality was found to be directly proportional to the concentration of test extracts when analyzed by using serial dilution method. Out of 14 organic extracts screened for cytotoxic activity, 21.42% of the extracts demonstrated LC_50_ value below 50 μg/ml and were categorized as highly cytotoxic while 57.14% of them were considered as moderately cytotoxic (LC_50_ value ≥50 but ≤200 μg/ml). The remaining 21.42% of the extracts had LC_50_ values >200 μg/ml and were considered to have a weak cytotoxic activity under the experimental conditions. The results from screening of *Q. dilatata* extracts against *A. salina* larvae are shown in Table 2. The positive control, doxorubicin demonstrated an LC_50_ 5.93 μg/ml. Among all the individual extracts CM extract was found to be the most cytotoxic exhibiting the LC_50_ 34.54 ± 0.16 μg/ml signifying the effectiveness of a moderately polar binary solvent system against a highly polar or non-polar solvent system. A working concentration of <1% in DMSO was employed to prepare test mixtures as it is a safer solvent in brine shrimp lethality test compared to others such as Tween 20 [50]. Brine shrimp lethality assay is a simple and high throughput cytotoxicity test of bioactive chemicals. It is based on the killing ability of test compounds of a simple zoological organism, the brine shrimps. *Artemia* nauplii are used extensively in research and toxicology due to the commercial availability of dried cysts from which live test material can be hatched at will. This assay draws extrapolations on the safety of the plant extracts and further illustrates trends of their biological activities. In bioactivity evaluation of the plant extracts by brine shrimp bioassay, an LC_50_ value of less than 1000 μg/ml is considered to be cytotoxic [51]. In our study, 100% of the screened extracts revealed LC_50_ values <1000 μg/ml suggesting the presence of cytotoxic bioactive compounds responsible for the observed deaths.

**Table 2:** Cytotoxicity and protein kinase inhibition of different solvent extracts of *Q. dilatata*

Samples	Brine shrimp cytotoxicity (μg/ml)		THP-1 cytotoxicity (μg/ml)		Hep G2 cytotoxicity (μg/ml)		Isolated lymphocytes		Protein kinase inhibition (μg/disc)		
	% mortality	LC_50_	% inhibition	IC_50_	% inhibition	IC_50_	% inhibition	IC_50_	Diameter (mm) at 100 μg/disc		MIC
	200		20		20		20		Clear zone	Bald zone	
NH	33.6 ± 2.5^*a*^	>200	22.32 ± 2.41^*c*^	> 20	46.73 ± 0.85 ^*a*^	>20	8.76 ± 1.12	>20	--	7 ± 0.15^*c*^	--
C	80.00 ± 0.49^*b*^	96.45 ± 0.98	14.24 ± 1.77^*a*^	> 20	43.20 ± 1.56 ^*a*^	>20	10.43 ± 2.56	>20	--	25 ± 0.2^*a*^	12.5
A	42.5 ± 0.94^*b*^	>200	82.43 ± 1.68	3.88 ± 0.53	41.20 ± 1.56 ^*a*^	>20	14.43 ± 1.21	>20	--	8 ± 0.47	--
EtA	80.0 ± 0.47^*a*^	77.7 ± 1.14	38.42 ± 2.13^*b*^	> 20	38.03 ± 2.28 ^*b*^	>20	12.43 ± 1.43	>20	--	7 ± 0.39^*c*^	--
Et	50.0 ± 1.25^*c*^	>200	47.25 ± 2.99	> 20	41.32 ^*a*^ ± 2.56 ^*b*^	>20	15.21 ± 1.22	>20	--	27 ± 0.47^*a*^	12.5
CE	91.5 ± 0.94 ^*c*^	49.62 ± 0.22	19.56 ± 3.22	> 20	9.72 ± 0.87	>20	13.87 ± 2.12	>20	--	9 ± 0.52^*b*^	--
CM	100.0 ± 1.70^*a*^	34.54 ± 0.16	21.56 ± 2.87	> 20	28.27 ± 0.85	>20	6.65 ± 1.54	>20	--	7 ± 0.21	--
EEt	100.0 ± 0.94	81.4 ± 0.17	43.12 ± 3.18	> 20	42.79 ± 0.76 ^*a*^	>20	5.86 ± 1.01	>20	--	9 ± 0.15	--
MEt	80.30 ± 2.36	69.9 ± 0.18	71.5 ± 4.11	5.59 ± 0.25	23.51 ± 1.54	>20	14.76 ± 2.22	>20	--	10 ± 0.41^*b*^	--
E	100.0 ± 0.94^*a*^	86.62 ± 1.35	82.52 ± 1.45^*b*^	4.95 ± 0.53	42.30 ± 1.12 ^*a*^	>20	12.87 ± 1.12	>20	--	26 ± 0.42^*a*^	12.5
DA	82.40 ± 1.25	49.1 ± 1.18	62.85 ± 2.45	9.24 ± 0.53	26.21 ± 1.21	>20	16.34 ± 4.45	>20	--	8 ± 0.35	--
M	91.50 ± 1.70	71.0 ± 0.74	22.53 ± 0.83^*a*^	> 20	45.50 ± 1.43 ^*a*^	>20	18.45 ± 3.98	>20	--	28 ± 0.35^*a*^	12.5
DM	80.50 ± 1.70	92.3 ± 1.70	42.54 ± 2.45	> 20	9.64 ± 0.76 ^*c*^	>20	22.54 ± 5.16	>20	--	8 ± 0.35	--
D	90.0 ± 1.70	78.4 ± 1.70	48.54 ± 0.83^*a*^	> 20	--	>20	12.11 ± 1.15	>20	--	9 ± 0.55	--
Doxorubicin	100	5.93			98 ± 0.18	5.1					
5-Florouracil			100	5							
Vincristine			100	8.1			73.45 ± 1.56	6.66 ± 0.22			
Surfactin										30 ± 1.02	
DMSO			--						--	--	
1% DMSO in PBS/sea water	--		--		--		--	--			

Values are presented as mean ± standard deviation of triplicate analysis. --: no activity. ^a-c^ means difference is highly significant, slightly significant, significant at *p* < 0.05

#### Cytotoxicity against THP-1 cell line

Advances in prevention and treatment of cancer requires continuous development of novel and improved chemotherapeutic and chemopreventive agents. The plant based anticancer drug discovery such as vincristine, vinblastine, etoposide, paclitaxel, camptothecin, topotecan and irinotecan accentuate the need for their further exploration as fundamental [52]. Keeping in view the prodigious cytotoxic potential illustrated by brine shrimp lethality assay; the plant extracts were further screened for an in vitro cytotoxic activity using human leukemia (THP-1 ATCC# TIB-202) cell line (Table 2). Samples were found to be more cytotoxic at higher concentrations as compared to lower concentrations. Among all the test extracts, most prominent inhibition was shown by A extract exhibiting 82.43 ± 1.68% inhibition at 20 μg/ml concentration and an IC_50_ 3.88 ± 0.53 μg/ml which is comparable to the standard drugs 5-florouracil and vincristine with IC_50_ 5 μg/ml and 8.1 μg/ml respectively. Previously methanol extract of *Q. dilatata* aerial parts demonstrated a noteworthy antitumor activity exhibiting 84.78% tumour inhibition at a concentration of 1000 μg/ml in potato disc antitumor assay [53]. Previously water infusions of mature and fresh *Q. resinosa* leaves were evaluated for antioxidant activity and genotoxic effects on HeLa cells. Results showed that fresh leaves infusions increase the oxidative process and other damage to DNA and may serve as a potential source of phenolics with anticancer activity [54]. In the present analysis, there was no significant correlation observed between TPC and THP-1 cell line cytotoxicity (R^2^ = 0.0542) or TFC and THP-1 cell cytotoxicity (R^2^ = 0.3288).

#### Cytotoxicity against Hep G2 cell line

The cytotoxicity of *Q. dilatata* crude extracts against Hep G2 human hepatoma cell line was determined as percent inhibition at 20 μg/ml concentration in comparison to the anticancer drug doxorubicin (Table 2). A moderate antiproliferative activity was exhibited by 50% of the test extracts with IC_50_ 46.73 ± 0.85–38.03 ± 2.28%. The highest inhibition was exhibited by the non-polar NH extract (46.73 ± 0.85%) followed by M > C > EEt > E > Et > EtA > CM > DA > MEt > DM > A = D. Hepatoma is amongst the two major forms of primary liver cancers and is the most common widespread cancer in the world which is preceded by the occurrence of hepatocellular damage via ROS and the generation of chronic inflammation [55]. Therefore, the antioxidant stature and cytotoxicity of *Q. dilatata* extracts as revealed in the present exploration suggests it to be a worthy hit for the identification of lead compounds against hepatoma. In the present analysis, there was no significant correlation observed between TPC and Hep G2 cell cytotoxicity (R^2^ = 0.128) or TFC and Hep G2 cell cytotoxicity (R^2^ = 0.0542).

#### Cytotoxicity against isolated lymphocytes

Cytotoxicity of sample extracts was also tested against isolated lymphocytes at the same concentration employed for cytotoxicity assessment against THP-1 and Hep G2 cell lines i.e. 20 μg/ml to compare effect at same concentration (Table 2). The results showed that none of the extracts was cytotoxic against the isolated lymphocytes indicating selective response against cancer cell lines which is beneficial in targeting cancer cells while limiting damage to normal cells. Vincristine, the positive control employed in the assay exhibited significant cytotoxicity against normal lymphocytes with IC_50_ 6.66 ± 0.22 μg/ml. The selective inhibition of THP-1 and Hep G2 cells indicate prospective of *Q. dilatata* as candidate for anticancer drug development.

#### Protein kinase inhibition assay

The results of zones of protein kinase inhibition recorded for the test samples are presented in Table 2. A direct relationship was observed between concentrations and activity. Among all the extracts, a noteworthy inhibition zone of 28 ± 0.35 mm bald phenotype (MIC = 12.5 μg/ml) was formed around M extract loaded disc followed by Et (27 ± 0.47 mm bald) and E extracts (26 ± 0.42 mm bald). These results are comparable with 30 mm bald zone of surfactin. The non-toxic effect of DMSO (negative control) was confirmed by the absence of growth inhibition zone. In recent years, there has been a tremendous surge for the discovery of protein kinase enzyme inhibitors especially from plants. Protein phosphorylation at serine/threonine and tyrosine residues by protein kinases is one of the major mechanisms regulating biological processes including apoptosis, cell proliferation, cell differentiation, and metabolism. Deregulated phosphorylation at serine/threonine and tyrosine residues by protein kinases resulting from genetic alterations acquired early in tumorigenesis are often the cause of cancer. In this respect, inhibition of protein kinases has emerged as a promising target for cancer treatment [21]. Protein kinase activity is critical for the aerial hyphae formation of *Streptomyces* and this prerequisite was exploited in the present study as kinase inhibitory effect of extracts represents their possible anticancer potential.

### Antileishmanial potential

**Fig. 5:**
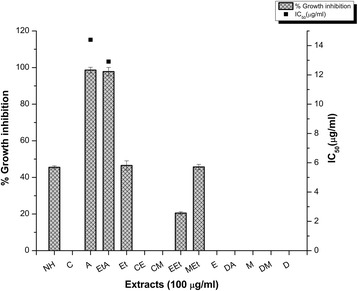
Antileishmanial potential of *Q. dilatata* extracts. Values are presented as mean ± Standard deviation from triplicate investigation. *IC_50_ > 100 μg/ml

Antileishmanial capability of different extracts of *Q. dilatata* has been evaluated for the first time in the current study. From the screening performed, EtA and A extracts exhibited remarkable and comparable leishmanicidic potential with IC_50_ 12.91 ± 0.02 μg/ml and 14.40 ± 0.01 respectively and it was found to be absent in the polar extracts (Fig. 5). Amphotericin B, the positive control exhibited IC_50_ 0.01 μg/ml. Although the antipromastigote activity of both the extracts is comparable but the percentage yield of A extract is more than EtA extract suggesting it as the most efficient solvent system for the reclamation of leishmanicidic potential of *Q. dilatata* in terms of yield and bioactivity. Leishmaniasis that represents a significant disease burden especially in the developing countries requires exploration of new less toxic chemotherapeutic agents due to variable effectiveness of the current treatments, their associated side effects and absence of any vaccine [56]. Previously this was confined to northern sphere of Pakistan but now it is widely spreading throughout the country with cutaneous and visceral leishmaniasis being the major threats [57]. The World Health Organization advocates the use of traditional medicine for the treatment of tropical diseases such as leishmaniasis due to their established safe use in humans [58]. Previously, *Q. insignis* ethanol bark extract showed promising activity against *L. amazonensis* promastigotes with low cytotoxic activity and it was suggested as a worthy candidate for further phytochemical exploration [2].

In the present study the C extract showed profound kinase inhibitory activity, cytotoxicity against shrimps and antileishmanial activity but did not exhibit any significant anti-proliferative potential against THP-1 or Hep G2 cell lines. The observed lethality against shrimps and promastigotes might be due to the inhibition of various kinases mandatory for cell survival. On the other hand among all the analyzed samples, E extract displayed significant cytotoxicity against shrimps and THP-1 leukemia cell line as well as protein kinase inhibition; therefore, mechanistic studies are required to extrapolate these activities for its possible anticancer role. In the present analysis, there was no significant correlation observed between TPC and antipromastigote activity (R^2^ = 0.0077) or TFC and antileishmanial potential (R^2^ = 0.0165).

### α-Amylase inhibition assay

**Fig. 6:**
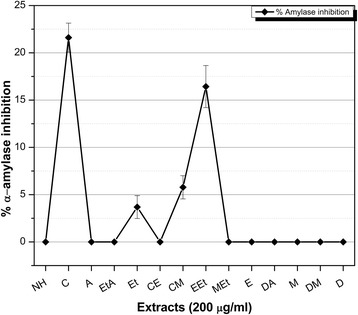
α**-**amylase inhibition by *Q. dilatata* extracts. The IC_50_ of acarbose (positive control) = 33.73 ± 0.12 μg/ml. Experiment was performed in triplicate and values are presented as mean ± standard deviation

All the fourteen extracts of varying polarities prepared from *Q. dilatata* were subjected to standard chromogenic α-amylase inhibition assay and the results are presented in Fig. 6. In our present study, C, EEt, CM and Et extracts were found to have some acarbose like antidiabetic activity i.e. 21.61 ± 1.53, 16.43 ± 2.23, 5.78 ± 1.23 and 3.69 ± 1.21% respectively. IC_50_ values were not calculated due to comparatively moderate inhibition (<50%) of subject enzyme. Acarbose, the positive control inhibited 80.34 ± 1.12% of α-amylase enzyme’s activity and demonstrated an IC_50_ 33.73 ± 0.12 μg/ml. The hallmark in diabetes control is the management of blood glucose level which may be achieved through the use of oral hypoglycaemic agents, insulin secretagogues, and carbohydrate hydrolysing enzyme inhibitors. Plants continue to play an important role in the treatment of diabetes, particularly in developing countries where most of the people have limited resources and do not have access to modern treatment. Even in the developed countries there is a shift to the use of alternative approaches to treat diabetes, such as plant-based medicines owing to the side effects associated with the use of insulin and oral hypoglycaemic agents [59]. Therefore, it is crucial to identify and explore the inhibitors of carbohydrate hydrolysing enzymes such as α-amylase from natural sources having fewer side effects. Inhibitors of α-amylase reduce the glucose peaks that can occur after a meal, slowing the speed with which amylase can convert starch to simple sugars until the body can deal with it. This is of particular importance in people with diabetes, where low insulin levels prevent extracellular glucose from being cleared quickly from the blood. Previous studies on α-amylase inhibitors isolated from medicinal plants suggest that several potential inhibitors of this enzyme belong to flavonoid class of phytochemicals [60]. However in the present analysis there was no significant correlation observed between TPC and amylase inhibition (R^2^ = 0.258) or TFC and enzyme inhibition (R^2^ = 0.258).

### Antifungal assay

The plant’s antifungal potential was assessed against four strains of filamentous fungi, the results of which are summarized in Table 3. The data indicate that a moderate antifungal activity was exhibited by almost all the extracts against the tested strains. In case of *A. fumigatus* the growth inhibition zones ranged between 7 and 8 mm for all the extracts. A maximum inhibition zone was displayed by the M, D and E extracts against *F. solani* (9 ± 1.54 mm), *A. niger* (9 ± 0.75 mm) and *A. flavus* (9 ± 0.98 mm) respectively. The absence of growth inhibition zone around negative control disc confirmed the non-toxic effect of DMSO whereas standard drug Clotrimazole exhibited maximum activity at a concentration of 10 μg/disc. It was observed that extraction solvent polarity did not profoundly affect antifungal proficiency of various *Q. dilatata* extracts. Since, well known plant secondary metabolites exhibiting antifungal activity include flavonoids, phenols and phenolic glycosides, unsaturated lactones, sulphur compounds, saponins, cyanogenic glycosides and glucosinolates [61].

**Table 3:** Antifungal activity of *Q. dilatata* extracts tested against filamentous fungi

Samples	^a^Diameter of growth inhibition zone at 100 μg/disc			
	*A. fumigatus*	*F. Solani*	*A. niger*	*A. flavus*
NH	7 ± 0.24	8 ± 1.85	7 ± 1.21	7.5 ± 0.45
C	7.5 ± 1.0	7 ± 0.25	7.5 ± 1.2	7.5 ± 0.25
A	7.5 ± 0.56	7.5 ± 1.21	7.5 ± 1.5	7.5 ± 0.32
EtA	7 ± 0.45	8 ± 0.56	7.5 ± 0.78	6.5 ± 0.41
Et	8 ± 0.52	8 ± 1.40	7 ± 0.25	8 ± 0.35
CE	7.5 ± 0.45	7 ± 0.35	8 ± 0.50	7.5 ± 0.30
CM	7 ± 0.35	8 ± 0.40	7 ± 0.57	7 ± 0.25
EEt	7.5 ± 1.2	7 ± 0.35	7.5 ± 0.20	7 ± 0.65
MEt	8 ± 0.40	7 ± 0.65	8 ± 1.10	7.5 ± 0.67
E	8 ± 0.45	7.5 ± 1.43	8 ± 1.0	9 ± 0.98
DA	7 ± 0.40	7 ± 1.50	7 ± 1.67	8 ± 0.55
M	7 ± 0.50	9 ± 1.54	8 ± 0.76	7.5 ± 0.69
DM	7.5 ± 0.5	8 ± 0.78	7.5 ± 0.80	6.5 ± 0.55
D	8 ± 0.55	7.5 ± 0.45	9 ± 0.75	7 ± 0.40
Clotrimazole	23 ± 0.12	24 ± 0.98	22 ± 1.03	24 ± 1.01
DMSO	--	--	--	--

^a^Zone of inhibition including the diameter of disc (6 mm). In each disc, the sample size was 100 μg per disc (5 μl) in disc diffusion assay. --: no activity. Values are presented as mean ± standard error from triplicate investigation

## Conclusion

The quantification of important polyphenols and determination of antimicrobial activity in various crude extracts of *Q. dilatata* is helpful in explaining some of its traditional uses. The use of a wide-ranging solvent system polarity proved crucial to demonstrate phytochemical and biological profiling of the subject plant**.** The current study proposes DA extract of *Q. dilatata* as a potential source of phytochemicals with substantial antioxidant capability while its M extract has protective activity against various tumorigenic kinases. Similarly, C extract was significantly cytotoxic against brine shrimps, THP-1 human leukemia cells and leishmanial promastigotes. In conclusion, the present study endorses use of polarity dependent extraction as an important factor for the determination of biological spectrum of *Q. dilatata* and prospects it as substantial source of bioactive metabolites.

## Abbreviations

DMSO: Dimethyl sulfoxide
DPPH: 2, 2-diphenyl-1-picrylhydrazyl
FRSA: Free radical scavenging activity
IC_50_: 50% inhibitory concentration
LC_50_: Lethal concentration causing 50% mortality
MIC: Minimum inhibitory concentration
ROS: Reactive oxygen species
TAC: Total antioxidant capacity
TFC: Total flavonoid contents
TPC: Total phenolic contents
TRP: Total reducing power
ZOI: Zone of inhibition
